# Basic and clinical study of efficacy and adverse effects of flumatinib in Ph^+^ ALL

**DOI:** 10.3389/fphar.2023.1178393

**Published:** 2023-05-05

**Authors:** Jun Wang, Jiafei Wu, Yijing Wang, Boyue Zheng, Yu Wang, Chuanyan Jiang, Mengying Zou, Hui Li

**Affiliations:** ^1^ Department of Hematology, Sichuan Provincial People’s Hospital, University of Electronic Science and Technology of China, Chengdu, China; ^2^ School of Clinical Medicine, Chengdu Medical College, Chengdu, China; ^3^ School of Clinical Medicine, University of Electronic Science and Technology of China, Chengdu, China; ^4^ Department of Hematology, Chengdu Second People’s Hospital, Chengdu, China; ^5^ Department of Hematology, Chengdu BOE Hospital, Chengdu, China

**Keywords:** philadelphia chromosome-positive acute lymphoblastic leukemia, flumatinib, treatment, new diagnosis, effect

## Abstract

**Objective:** To investigate the efficacy and safety of chemotherapy in treating Ph+ ALL based on flumatinib.

**Methods**: The clinical data of 29 patients with Ph+ ALL receiving flumatinib-based chemotherapy in Sichuan Provincial People’s Hospital from January 2020 to January 2023 were collected for analysis, with the concentrations of TKI in the peripheral blood, bone marrow, and cerebrospinal fluid of some patients monitored, Cytological experiments on SUP-B15 were conducted in a Ph+ ALL cell line.

**Results:** A total of 29 patients were enrolled, showing the induced CR, 3-month CR, and 6-month CR rates of 96.3%, 87.5%, and 86.7%, respectively after flumatinib-based chemotherapy. The negative conversion ratio of MRD was 82.6%, 91.3%, and 95.6% in 1, 2, and 3 months after treatment, respectively, with 4.3% of patients failing the conversion in 3 months after treatment. The rates of MMR were 73.9%, 87.5%, and 93.3% in 1, 3, and 6 months after treatment, and CMR of 52.2%, 62.5%, and 73.3%, respectively. Among the 29 patients, 11 (37.9%) received transplant and the continuous flumatinib for 1 year after transplantation. The deep remission was maintained in all patients up to the time of follow-up, with the median follow-up of 12 months (1–33 months), progression-free survival (PFS) of 11 months (1–33 months), and median overall survival (OS) of 12 months (1–33 months). The adverse reactions mainly referred to myelosuppression, liver insufficiency and infection that were generally tolerable. In terms of blood concentration, the concentration of flumatinib was ordered as bone marrow > serum > cerebrospinal fluid in Ph+ ALL bone marrow. In contrast, the concentration of dasatinib and imatinib was ordered as serum > bone marrow > cerebrospinal fluid. At the same time, flumatinib has a high probability to cross the blood-brain barrier, while the concentration of cerebrospinal fluid in the patients using Dasatinib was lower compared to the lower limit of detection in this study. Compared with Imatinib and Dasatinib, flumatinib exerted the most potent inhibitory effect on Ph+ ALL cell lines according to pharmacodynamic analysis of SUP-B15 cells.

**Conclusion:** Flumatinib combined with chemotherapy could achieve good efficacy and safety in treating Ph+ ALL, with flumatinib in a high probability of crossing the blood-brain barrier. Flumatinib could be a superior choice to Dasatinib and Imatinib in cell experiments.

## 1 Introduction

Philadelphia chromosome-positive acute lymphoblastic leukemia (Ph+ ALL) is a common type of acute lymphoblastic leukemia (ALL), with the banded analysis of t (9; 22) (q34; q11), accounting for about a quarter of ALL adults (Horowitz et al., 2018), typically characterized by aggressive progression, poor response to standard chemotherapy, and a high risk of recurrence (Yilmaz et al., 2018). The application of TKI combined with chemotherapy significantly elevated the 5-year overall survival rate of Ph+ ALL from 10% to 40%–50% (Soverini et al., 2019). Since the first generation of TKI Imatinib, the second generation of Dasatinib, Nilotinib, and other drugs have gradually served to treat Ph+ ALL patients, leading to significant progress in Ph+ All-induced remission. However, a lot of adverse reactions have also occurred during TKI preparation (Zhu and Qian, 2014; Brattås et al., 2019). The dose of medication is forced to be reduced or even stopped in some patients due to the severe adverse reactions. At the same time, with the extended TKI application time, many patients develop drug resistance, with the ABL kinase region mutation as the most common cause (Ma et al., 2019; Yang et al., 2019). In terms of chronic myeloid leukemia (CML), flumatinib, a second-generation TKI independently developed in China, could achieve a more considerable efficacy compared to imatinib, comparable to other second-generation TKI, resulting in fewer adverse reactions and higher safety, making it advantageous over other TKI preparations (Xue et al., 2021). However, there lack reports on clinical efficacy and adverse reactions of flumatinib in Ph+ ALL, especially on whether flumatinib can prevent central leukemia. Herein, the data of Ph+ ALL patients administrated by flumatinib in our center were retrospectively analyzed, with the relationship between the blood concentration of flumatinib in different parts (peripheral blood, bone marrow, and cerebrospinal fluid) and clinical efficacy and adverse reactions of the disease explored, to provide clinical reference for choosing reasonable TKI for Ph+ ALL patients.

## 2 Included cases and methods

### 2.1 Included cases

Clinical data of 29 patients with Ph+ ALL receiving flumatinib treatment in Sichuan Provincial People’s Hospital from January 2020 to January 2023 were collected for the retrospective analysis, all of whom had passed ethical review.

#### 2.1.1 Inclusion criteria

① Diagnosed as Ph+ ALL according to the NCCN guidelines; ② Age ≥ 18 years old; ③ Sign informed consent; ④ Administrated with flumatinib for at least 1 month.

#### 2.1.2 Exclusion criteria

① Age < 18 years old; ② Pregnant, preparing for pregnancy or lactating women; ③ Patients with secondary myelodysplastic syndrome (MDS), chronic myeloproliferative disease (MPN) and lymphoproliferative disease. ④ CML-LBC. ⑤ Malignant tumors with other progression; ⑥ Serious heart, liver, kidney organ insufficiency.

Baseline data of blood routine, blood biochemistry, primitive + infantile lymphocyte count in newly diagnosed bone marrow cytology, flow cytometry, fusion gene, BCR-ABL fusion gene quantification, all-related gene mutation, chromosome, ABL kinase region mutation site, and hepatitis B and C liver screening were recorded. The followed-ups were carried out for some of the above indexes throughout the treatment.

### 2.2 Treatment methods

(1) tyrosine kinase inhibitors: Patients are instructed to use tyrosine kinase inhibitors as soon as possible after diagnosed.
(2) Combination chemotherapy: all patients were treated with VP (Vincristine/Vindesine + prednisone) induction regimen in consolidation therapy, where 15 received Hyper-CVAD B (Methotrexate + Cytarabine + methylprednisolone) for consolidation therapy, and 5 for Hyper-CVAD A (cyclophosphamide + vincristine + doxorubicin/pirarubicin + dexamethasone), 2 for CAM (cyclophosphamide + cytarabine + 6-mercaptopurine), 1 (DDLD) for (dexamethasone + Pegaspargase + Nordoxorubicin + vindesine), and 1 for VP (Vinchnestine/vindesine + prednisone), two patients underwent induction therapy without consolidation, and three patients were lost during follow-up. Some patients were brided with blinatumomab, followed by allogeneic hematopoietic stem cell transplantation (allo-HSCT).
(3) Three intrathecal injections of methotrexate, cytarabine, and dexamethasone were performed for at least six times during treatment to prevent central nervous system leukemia.

**FIGURE 1:**
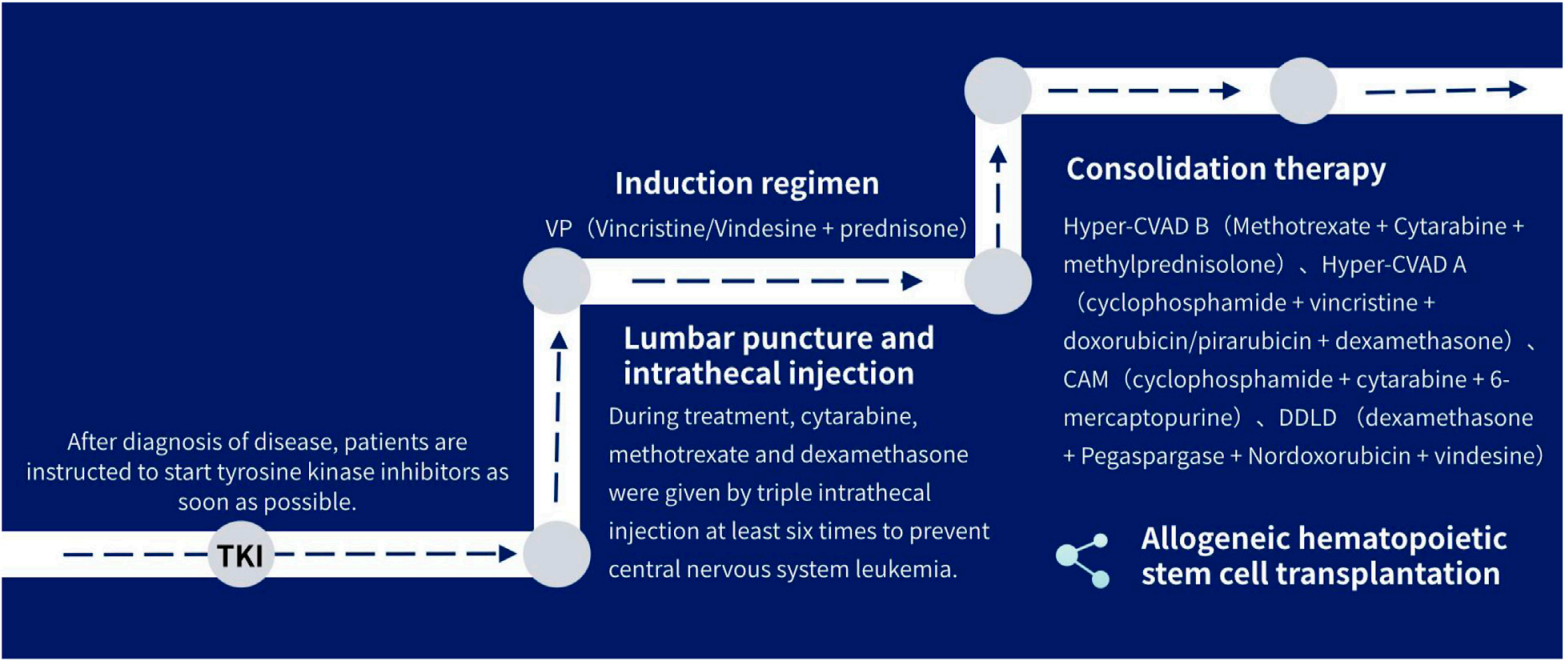
Treatment flow chart.

The treatment process is shown in Figure 1.

### 2.3 Efficacy evaluation and adverse reactions

The efficacy of ALL was evaluated based on the criteria of the National Comprehensive Cancer Network (NCCN), primarily for complete response rate (CR) and molecular response (MMR and CMR). Here, MRD levels in bone marrow and cerebrospinal fluid were detected using multi-parameter flow cytometry, with the second generation gene sequencing and the decline of BCR-ABL gene monitored.

The follow-up including outpatient, inpatient was carried out by telephone until 1 January 2023, with the median follow-up time of 12 months (1–33 months). Overall survival (OS) was defined as the time between initiation of flumatinib and the last follow-up or death, progression-free survival (PFS) time as the time from the start of flumatinib treatment until a change in the patient’s disease, such as disease progression or death.

Adverse reactions were evaluated using the National Cancer Institute (NCI) Standard for Evaluating Common Adverse Reaction Terms Version 5.0 (CTCAE V5.0).

### 2.4 Timing of TKI concentration detection

The trough concentration of TKI was detected in 1, 2, and 3 months after treatment. Samples of all patients were collected 30 min ahead of medication on the same day, with 2 mL of peripheral blood, bone marrow, and cerebrospinal fluid samples collected for each patient. Flumatinib blood-brain barrier permeability = concentration of flumatinib in cerebrospinal fluid/concentration of serum samples collected simultaneously.

### 2.5 Cytology experiment

#### 2.5.1 Cell proliferation detected by CCK-8 colorimetry

Logarithmic growth SUP-B15 cells were inoculated in a 96-well plate, with the concentration set at 6 × 105/L. The concentrations of the flumatinib were set as 25, 12.5, 6.25, 3.12, 1.56, and 0.78 μmol/L, respectively, 160, 80, 40, 20, 10, and 5 μmol/L for dasatinib, respectively, 40, 20, 10, 5, 2.5, and 1.25 μmol/L for imatinib, respectively. Wells of zero setting and blank control were set, with 3 multiple wells in each group and incubated in 37°C, 5% CO2 incubator for 24 h. CCK-8 solution of 20 μL was added to each well and incubated for 4 h. An enzyme-labeled meter was employed to detect the absorbance.

#### 2.5.2 Wright-giemsa staining method

SUP-B15 cells of logarithmic growth stage were inoculated in a 24-well plate, with the concentrations of 1.56, 0.78, 0.39 μmol/L for flumatinib, 10, 5, 2.5 μmol/L for dasatinib, and 20, 10, 5 μmol/L for imatinib, respectively. Three multiple wells were set and incubated in a 5% CO2 incubator at 37°C for 24 h. After incubation, the cell suspension was collected for centrifugation, then the supernatant was discarded. The smear was re-suspended with 100 μL PBS, fixed with methanol for 10 min, rinsed with PBS and dried, stained with Garrey-Giemsa at working solution for 30 min, rinsed with UP water and dried. Cell morphology was observed and photographed using an inverted microscope.

#### 2.5.3 The effect of three drugs on SUP-B15 cell apoptosis was detected by flow cytometry

SUP-B15 cells of logarithmic growth stage were inoculated in 24-well plate, with the cell density adjusted to 1 × 106/L, the concentrations of flumatinib were set as 1.56 and 0.78 μmol/L, 10 and 5 μmol/L for dasatinib, and 20 and 10 μmol/L for imatinib, respectively. Three multiple wells were set up in both groups, then incubated at 37°C in 5% CO2 incubator for 24 h. The supernatant was removed after centrifugation at 4°C, washed twice with PBS, removed after centrifugation, and stained with AnnexinV-EGFP/PI double labeling. The outcomes were determined by flow cytometry within 1 h.

#### 2.5.4 The effect of three drugs on the SUP-B15 cell cycle was detected by flow cytometry

SUP-B15 cells of logarithmic growth stage were inoculated into 24-well plates, with the cell density adjusted to 1 × 106/L. The concentrations of flumatinib were 3.12 and 1.56 μmol/L, respectively. Cells were collected, washed with PBS once, fixed after pre-cooled with 70% ethanol overnight, centrifuged to remove supernatant, washed with pre-cooled PBS, centrifuged to remove supernatant, added with a staining solution containing RNase A and PI, and detected by flow cytometry. Flow analysis was carried out on FlowJo software.

### 2.6 Statistical analysis

Statistical analysis was performed using GraphPad Prism version 9.4. The data were expressed as mean ± standard deviation or median. The Fisher exact probability test was performed when the total sample size was below to 40 or the minimum theoretical frequency below to 1. OS was evaluated by the Kaplan-Meier curve. The survival between groups was compared by log-rank test. Test level = 0.05, *p* < 0.05 was considered statistically significant.

## 3 Results

### 3.1 Baseline information of patients

A total of 29 patients with Ph+ ALL were enrolled in this study, with a median age of 47 (18–85) years, including 17 males (58.6%) and 12 females (41.4%), with 24 high-risk patients (82.8%). A total of 16 cases (55.1%) with hyperleukocytosis (white blood cell count >30*10^9^/L) were diagnosed for the first time. There obtained 19 patients (65.5%) with IKZF1 deletion mutation, 1 patient (3.4%) with RUNX1 gene mutation, 7 patients (24.1%) with negative gene mutations, and 3 patients (10.3%) refused gene testing. There categorized 22 (75.9%) P190-positive patients and 7 (24.1%) P210-positive patients in the BCR-ABL fusion genotype. There were 2 patients (6.9%) with HOX11 and 2 patients (6.9%) with WT1. According to genetic characteristics, 7 patients (27.6%) were normal in chromosome karyotype, 11 patients (37.9%) with Ph-positive alone, 6 patients (20.7%) with Ph-positive combined with other chromosome abnormalities, 1 patient (3.4%) with poor cell growth resulting in the failure to analyze the mitotic phase after multiple films, and 3 patients (10.3%) refused karyotype examination. Three patients (10.3%) had extramedullary infiltration, two (6.9%) with superficial lymph node enlargement, and one (3.4%) with arm mass.

Twenty-one patients (72.4%) received flumatinib after the first diagnosis, and 8 patients (27.6%) turned to flumatinib from other tyrosine kinase inhibitors. Among the 8 patients with drug change, 2 patients (25.0%) chose to change due to abnormal liver function, 2 patients (25.0%) due to renal insufficiency, and 1 patient (12.5%) due to liver and kidney insufficiency, 2 patients (25.0%) due to pleural effusion, and 1 patient (12.5%) due to drug resistance and mutation in the ABL kinase region.

### 3.2 Efficacy evaluation

#### 3.2.1 Overall efficacy

After the administration of flumatinib combined with VP induction, the induced CR, 3-month CR, and 6-month CR rates achieved 96.3%, 87.5%, and 86.7%, respectively. The negative conversion rate of MRD was 82.6%, 91.3%, and 95.6% in 1, 2, and 3 months after treatment, respectively, with 4.3% of patients showing no negative conversion. The rates of MMR and CMR were 73.9%, 87.5%, and 93.3%, and 52.2%, 62.5%, and 73.3% in 1, 3, and 6 months after treatment, respectively. Among the 29 patients, 11 (37.9%) were transplanted and continued to use flumatinib for 1 year after transplantation. The deep remission was maintained in all patients up to the time of follow-up. Median follow-up was 12 months (1–33 months), with progression-free survival (PFS) of 11 months (1–33 months), and median overall survival (OS) of 12 months (1–33 months) (Table 1).

**TABLE 1:** The efficacy of different treatment periods.

Remission	0.5 months	1 month	3 months	6 months
CR	84.2% (16/19)	96.3% (26/27)	87.5% (14/16)	86.7% (13/15)
MMR		73.9% (17/23)	87.5% (14/16)	93.3% (14/15)
CMR		52.2% (12/23)	62.5% (10/16)	73.3% (11/15)

#### 3.2.2 Comparison of curative effect between first-line and dressing change patients

**FIGURE 2:**
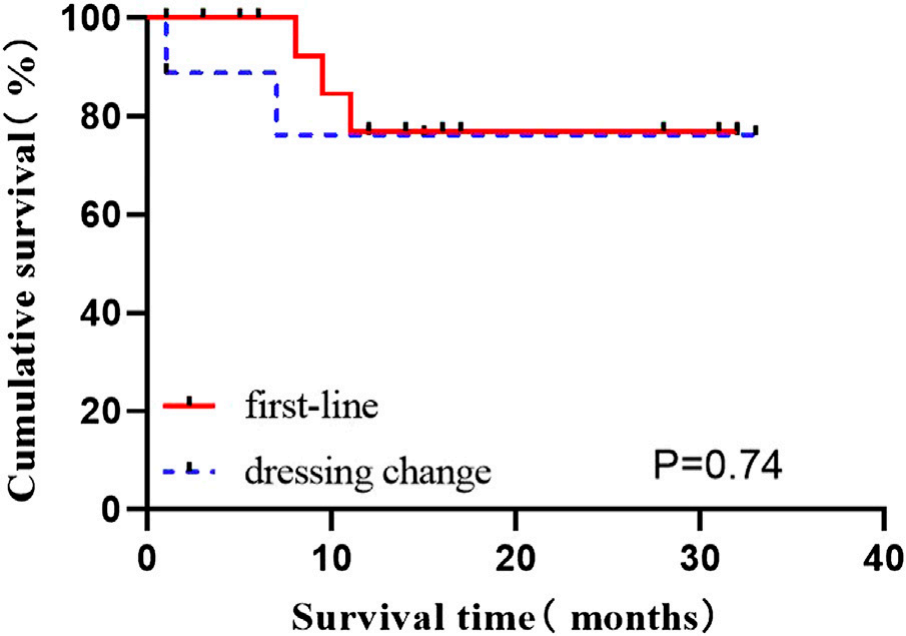
Effect of Effects of first-lineflumatinib and dressing change group on OS.

The data of induced response, MRD, and molecular response (CMR, MMR) in first-line patients newly diagnosed and those switched from other TKI to flumatinib were provided in Table 2. The OS status of two groups of patients were compared according to whether they were in remission after induction therapy, and revealed no statistical difference (all *p* > 0.05) (*p* = 0.074), suggesting no impact on long-term survival after switched to flumatinib (Figure 2).

**TABLE 2:** Efficacy of different groups.

Remission	First-line	Dressing change	*p*
Induced CR	94.7% (18/19)	100% (8/8)	1.000
1 month MRD	76.5% (13/17)	100% (6/6)	0.539
1 month MMR	68.8% (11/16)	85.7% (6/7)	0.621
1 month CMR	43.8% (7/16)	71.4% (5/7)	0.371
3 months MMR	83.3% (10/12)	100% (3/3)	1.000
3 months CMR	58.3% (7/12)	75.0% (3/4)	1.000
6 months MMR	90.0% (9/10)	100.0% (5/5)	1.000
6 months CMR	70.0% (7/10)	80.0% (4/5)	1.000

#### 3.2.3 Impact of age on OS

**FIGURE 3:**
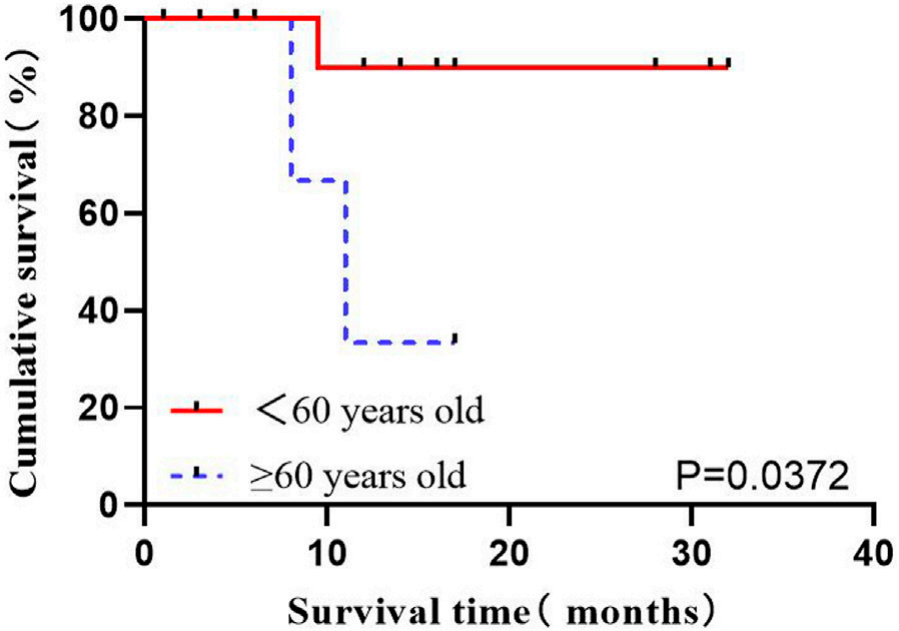
The influence of age stratification on OS.

Age is considered one of the critical factors affecting the prognosis of ALL. The data of a total of 21 patients directly receiving flumatinib at first diagnosis were analyzed, as divided into two age ranges: <60 years old and ≥60 years old. The influence of age on OS in Ph+ ALL patients was analyzed, which indicated an association of age over 60 (including 60) with the worse prognoses (*p* = 0.0372) (Figure 3).

#### 3.2.4 Impact of whether to merge IKZF1 on OS

**FIGURE 4:**
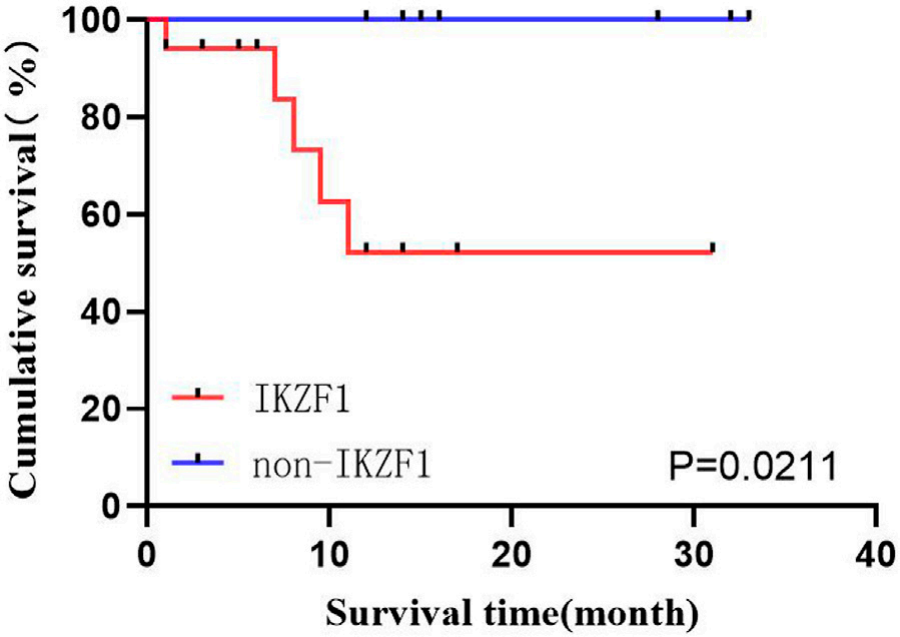
Effects of IKZF1 on OS.

Among the 29 patients, 19 (65.5%) exhibited IKZF1 deletion mutation, where 17 could receive follow-up, and 2 were lost. Ten patients exhibited no IKZF1 (34.5%), where 9 could receive follow-up, and 1 was lost. Survival analysis was performed using the Kaplan-Meier method in both groups, which revealed the worse prognoses in patients with IKZF1 (*p* = 0.0211) (Figure 4).

#### 3.2.5 Effects of the induced remission rate on OS

**FIGURE 5:**
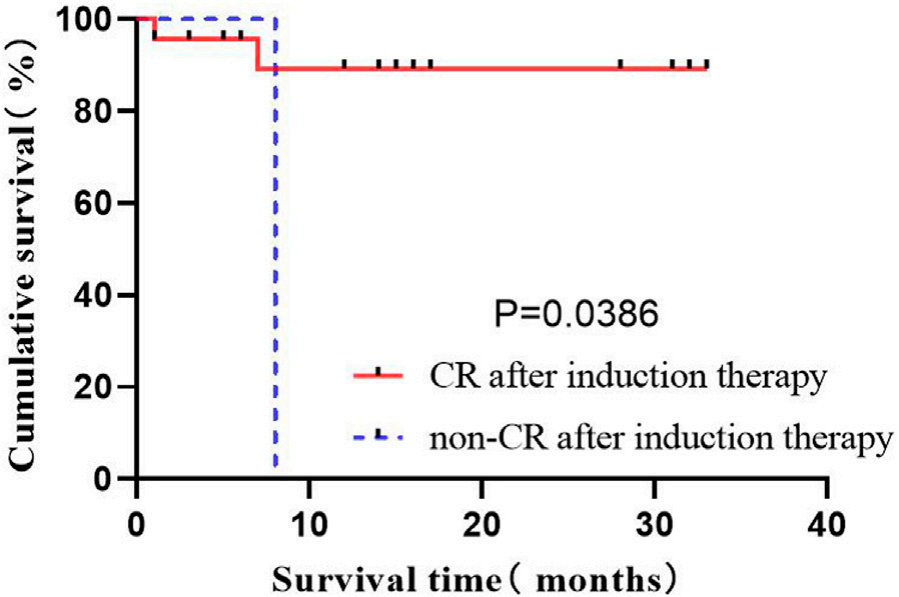
Effect of CR on OS after induction.

The complete remission of bone marrow is considered leukemia-free by morphology, as the basic condition to judge the effectiveness of treatment. In the present study, it covering 29 patients was evaluated after inducted with the flumatinib + VP regimen. A total of 26 patients obtained CR, and 1 patient did not respond. The population was divided to compare their OS status according to whether they were in remission after induction therapy, revealing the significantly prolonged OS of patients by complete remission after induction therapy (*p* = 0.0386) (Figure 5).

#### 3.2.6 Impact of MMR on OS after 3 months

**FIGURE 6:**
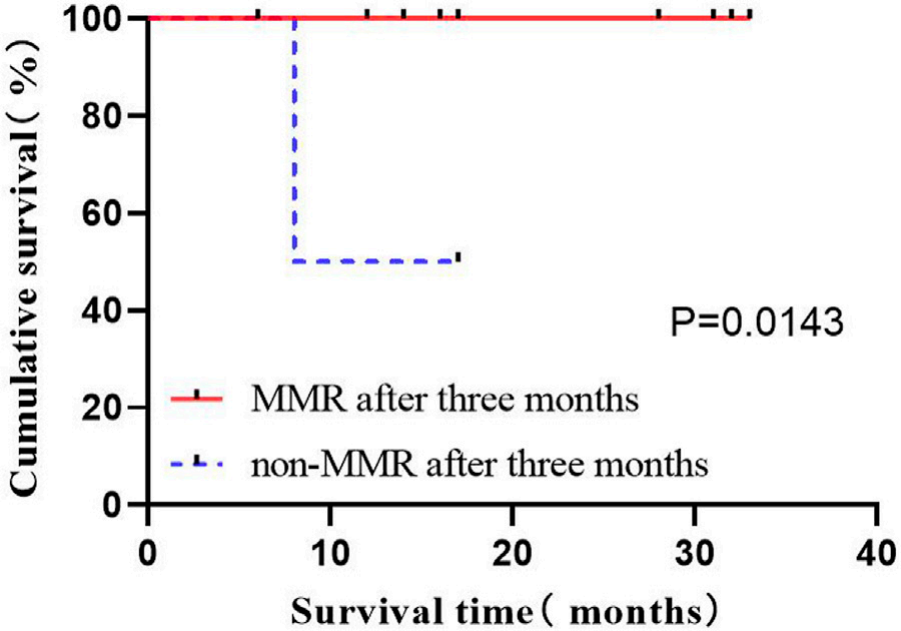
The effect of MMR on OS in 3 months.

Among the 29 patients, 16 patients (87.5%) had MMR, where 2 exhibited no MMR in 3 months after treatment. The study population was divided into groups according to whether they had achieved MMR 3 months after treatment to compare OS status. The results indicated the prolonged OS of patients by the implementation of MMR in 3 months after treatment (*p* = 0.0143) (Figure 6).

#### 3.2.7 Effects of hematopoietic stem cell transplantation on OS

**FIGURE 7:**
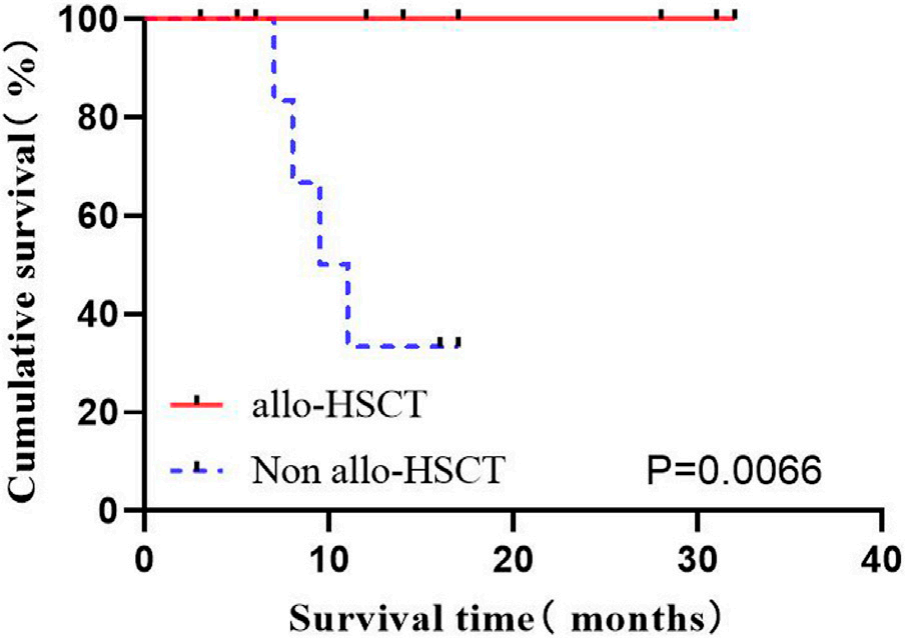
The effect of HSCT on OS.

Among the 29 cases, 11 (37.9%) underwent autoHCT after consolidation therapy, all maintained deep remission. However, some old patients did not choose to enter autoHCT considering the high risk, economic reasons, incompatibility and other reasons. A total of 20 patients were classified into the high-risk group with consolidation therapy, of which 8 were transplanted immediately. The high-risk group was divided into two groups according to whether chose transplant, to compare the OS of patients. The results showed the significantly prolonged OS by autoHCT (*p* = 0.0066) (Figure 7).

Survival analysis on the effects of sex, risk stratification, hyperleukocytosis at first diagnosis, different types of BCR-ABL, negative turn of MRD 1 month after treatment, and CMR in 3 months after treatment on OS was conducted, revealing no statistical difference (all *p* > 0.05).

### 3.3 Evaluation of adverse reactions

The main adverse effects of flumatinib referred to myelosuppression, with 4 cases (16.7%) of grade II myelosuppression, 2 (8.3%) of grade III myelosuppression, and 18 (75.0%) of grade IV myelosuppression. The median minimum values for neutropenia were 0.15 (0–1.79) ×10^9/L, 56 (0–59) ×10^9/L for hemoglobin, and 21 (0–51) ×10^9/L for platelets. Most of the non-hematological adverse reactions were liver insufficiency, of which 6 patients (50.0%) had grade I liver function impairment, 3 patients (25.0%) with grade II liver function impairment, and 3 patients (25.0%) with grade III liver function impairment. The liver function of the above patients returned to normal after receiving symptomatic treatment, such as the stabilized liver cell membrane.

### 3.4 Drug concentration monitoring

After 1 month of flumatinib in Ph+ ALL patients, the concentrations of flumatinib in serum, bone marrow, and cerebrospinal fluid reached 32.5 ± 24.4 ng/mL (12–94 ng/mL), 128.9 ± 81.2 ng/mL (41.3–318 ng/mL) and 0.67 ± 0.61 ng/mL (0–1.5 ng/mL), respectively. The blood-brain barrier permeability of flumatinib was 3.01% ± 3.55% (0–8.48%). Two months after flumatinib, the concentration of flumatinib in serum and bone marrow reached 21 ng/mL and 35.4 ng/mL, respectively. After 3 months, the concentrations in serum, bone marrow, and cerebrospinal fluid were 46.22 ± 20.93 ng/mL (19–77 ng/mL), 148.9 ± 100.05 ng/mL (62.5–290 ng/mL), and 0.57 ± 0.43 ng/mL (0.3–1.2 ng/mL), respectively. The blood-brain barrier permeability of flumatinib was calculated as 1.39% ± 0.87% (0.39%–2.14%), with the concentrations of flumatinib in serum, bone marrow, and cerebrospinal fluid of 34.37 ± 14.53 ng/mL (25–51.1 ng/mL) and 119 ± 90.51 ng/mL (55–183 ng/mL), respectively, and 1.5 ng/mL, respectively. The blood-brain barrier penetration rate of flumatinib was calculated as 6%. Flumatinib concentrations in serum, bone marrow, and cerebrospinal fluid were 80.4 ng/mL, 203 ng/mL, and 0.9 ng/mL, respectively after 5 months, with the blood-brain barrier permeability as 1.12%. After 10 months, the concentrations in serum, bone marrow, and cerebrospinal fluid reached 38, 214, and 0.36 ng/mL, respectively, with the blood-brain barrier permeability of flumatinib as 0.95%. After 1 year, the concentrations in serum, bone marrow, and cerebrospinal fluid were 29.93 ± 9.09 ng/mL (19.8–41.9 ng/mL), 59.75 ± 21.71 ng/mL (44.4–75.1 ng/mL), and 0.9 ± 0.17 ng/mL (0.7–1 ng/mL), with the blood-brain barrier permeability as 2.86% ± 1.03% (1.67%–3.45%) (Table 3).

**TABLE 3:** Determination of flumatinib concentration at different time points.

Flumatinib drug **concentr tion**	One month	Two months	Four months	Five months (%)	Ten months (%)	After 1 year
Cerebrospinalfluid/plasma	3.01% ± 3.55% (0–8.48%)	1.39% ± 0.87% (0.39–2.14%)	6.00%	1.12	0.95	2.86% ± 1.03% (1.67%–3.45%)
Bone marrow/plasma	441.57% ± 217.84% (100–709.9%)	305.44% ± 91.05% (202.85–376.62%)	448.89% ± 323.70% (220.00–677.78%)	252.49	563.16	201.74% ± 31.82% (179.24%–224.24%)

The concentrations of dasatinib in 3 Ph+ ALL patients and imatinib in 2 Ph+ ALL patients were also determined, the cerebrospinal fluid was <1 ng/mL in all. Distinguished from the patients using flumatinib, the concentration of drugs in those using dasatinib was higher compared to bone marrow. The detection was conducted in 3, 5, 7, and 9 months after the use of dasatinib, respectively. The concentrations in serum was 6.2 ± 3.41 ng/mL (3–11 ng/mL) and 3.23 ± 0.40 ng/mL (3–3.7 ng/mL) in bone marrow. Two patients with Ph+ ALL treated with imatinib for 1 month exhibited the serum imatinib concentration of 3,320 ± 183.85 ng/mL (3,190–3,450 ng/mL) and bone marrow Imatinib concentration of 3,020 ± 127.28 ng/mL (2,930–3,450 ng/mL). The concentration of imatinib in cerebrospinal fluid was 34 ng/mL in one patient. The blood-brain barrier penetration rate of imatinib was 1.07%.

### 3.5 Cytology experiment

#### 3.5.1 CCK-8 experiment

**FIGURE 8:**
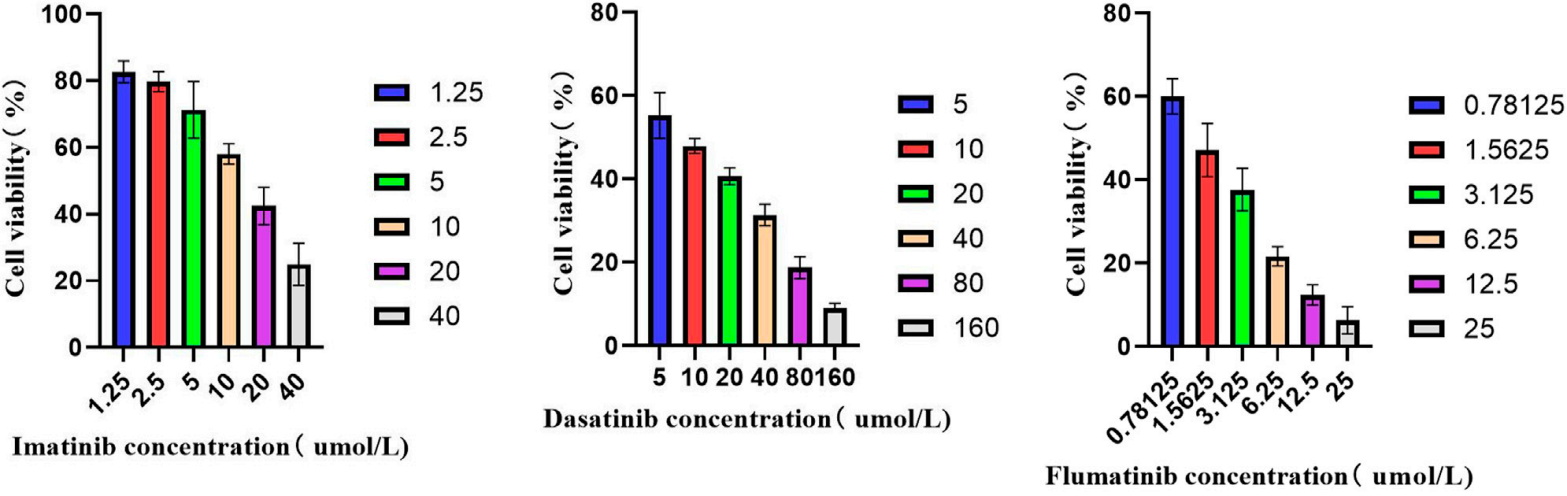
The activity of SUP-B15 cells under different administration concentrations of Imatinib, Dasatinib, and flumatinib.

Firstly, the effects of imatinib, dasatinib, and flumatinib on SUP-B15 cell proliferation were detected, which all exhibited the dose—and time-dependent inhibitory effects on SUP-B15 cell proliferation. The IC50 values of SUP-B15 cells treated with three drugs were 13.25, 8.63, and 1.388 μmol/L (Table 4), respectively, indicating a stronger inhibitory effect of flumatinib on SUP-B15 cell proliferation (Figure 8).

**TABLE 4:** IC50 values of different drugs for SUP-B15 cell.

TKI	Imatinib	Dasatinib	Flumatinib
IC_50_(μmol/L)	13.25	8.63	1.388

#### 3.5.2 Wright-giemsa staining was used to observe the effects of drug treatment for 24 h on cell morphology and number

**FIGURE 9:**
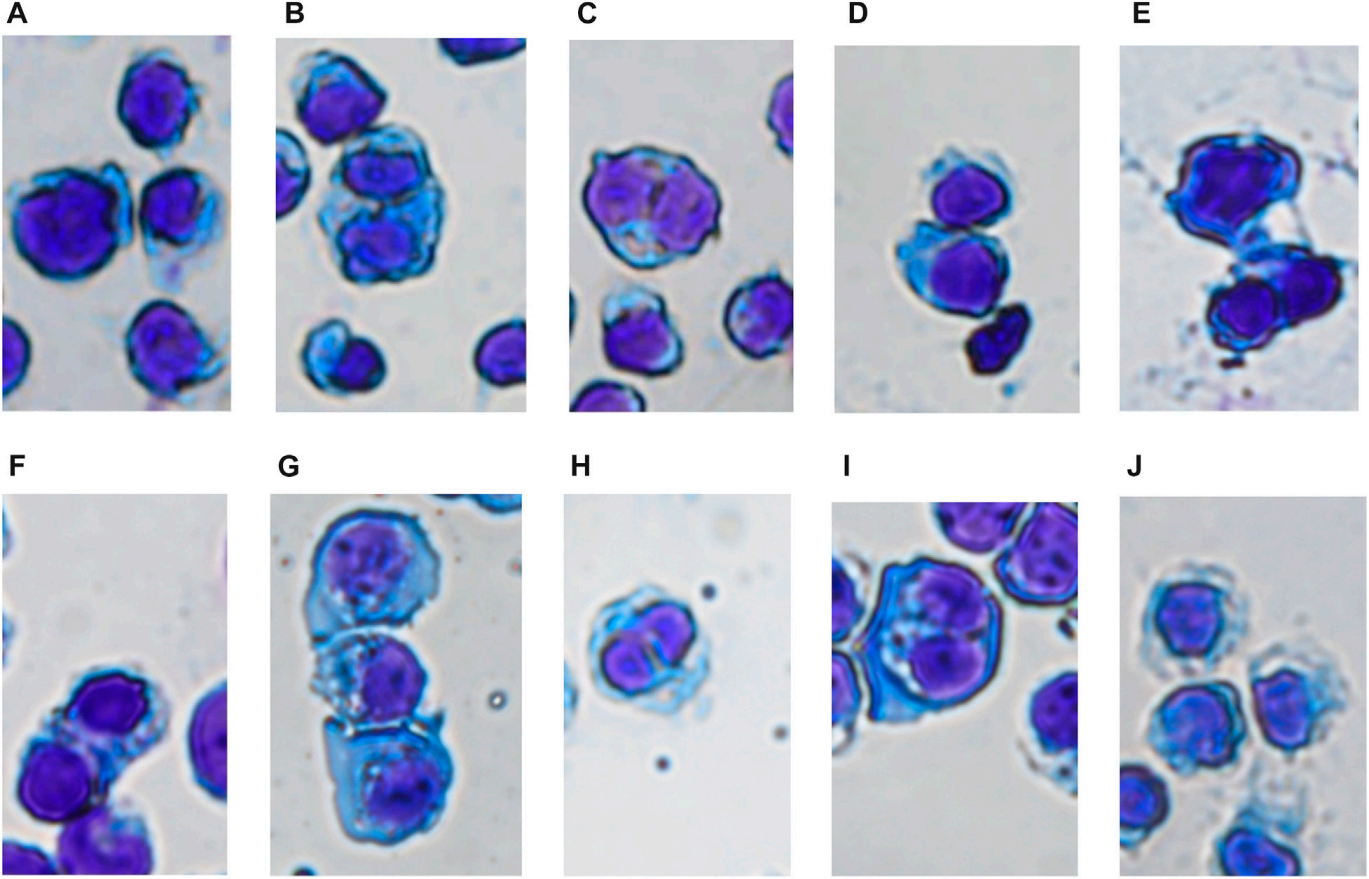
**(A–J)** Were the cell morphology of SUP-B15 cells under different administration concentrations of Imatinib, Dasatinib, and Flumatinib **(A)** Negative control group; **(B)** Imatinib 20 μmol/L; **(C)** Imatinib 10 μmol/L; **(D)** Imatinib 5 μmol/L; **(E)** Dasatinib 10 μmol/L; **(F)** Dasatinib 5 μmol/L; **(G)** Dasatinib 2.5 μmol/L; **(H)** Flumatinib 1.56 μmol/L; **(I)** Flumatinib 0.78 μmol/L; **(J)** Flumatinib 0.39 μmol/L.

In the control group, the nucleus of SUP-B15 was round with complete structure. The proportion of nucleus and nuclear cytoplasm was large. The cellular chromatin was abundant, indicating that the cell replication and proliferation ability were strong. In the TKI treatment group, the nuclear substance was significantly reduced, and the nuclear denaturation and shrinkage were observed. With the increase of drug concentration, the degree of nuclear shrinkage was greater, and the cytoplasm was gray blue. It is indicated that these three drugs can inhibit the proliferation of SUP B15 cell line in a dose-dependent manner (Figure 9).

#### 3.5.3 Flow cytometry plus annexin V-EGFP/propidium iodide (PI) double staining was used to analyze the effect of drug on apoptosis

**FIGURE 10:**
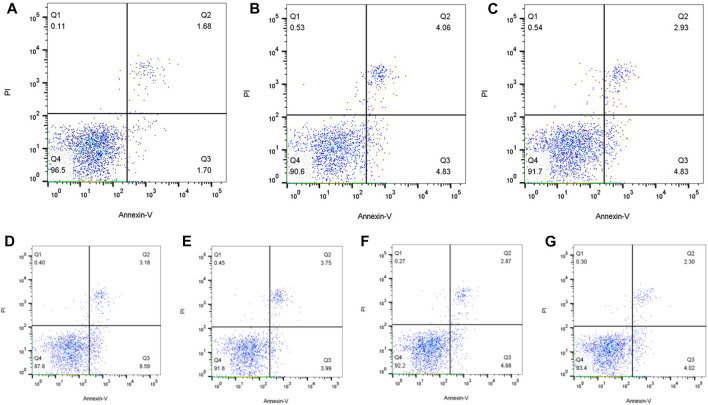
**(A–G)** Were the apoptosis of SUP-B15 cells under different administration concentrations of Imatinib, Dasatinib, and flumatinib **(A)** Negative control group; **(B)** Flumatinib 1.56 μmol/L; **(C)** Flumatinib 0.78 μmol/L; **(D)** Imatinib 20 μmol/L; **(E)** Imatinib 10 μmol/L; **(F)** Dasatinib 10 μmol/L; **(G)** Dasatinib 5 μmol/L.

Annexin V/PI double staining was employed for apoptosis detection, which was detected as 8.89% and 7.76% after treated with 1.56 μmol/L and 0.78 μmol/L flurmatinib for 24 h, 7.55% and 6.32% with 10 μmol/L and 5 μmol/L dasatinib for 24 h, and 11.77% and 7.74% with 20 μmol/L and 10 μmol/L imatinib for 24 h. The results indicated that TKI with different concentrations could promote the apoptosis of SUP-B15 cells, with a positive dose-dependence (Figure 10).

#### 3.5.4 The effect of drugs on cell cycle was analyzed by flow cytometry + PI staining

**FIGURE 11:**
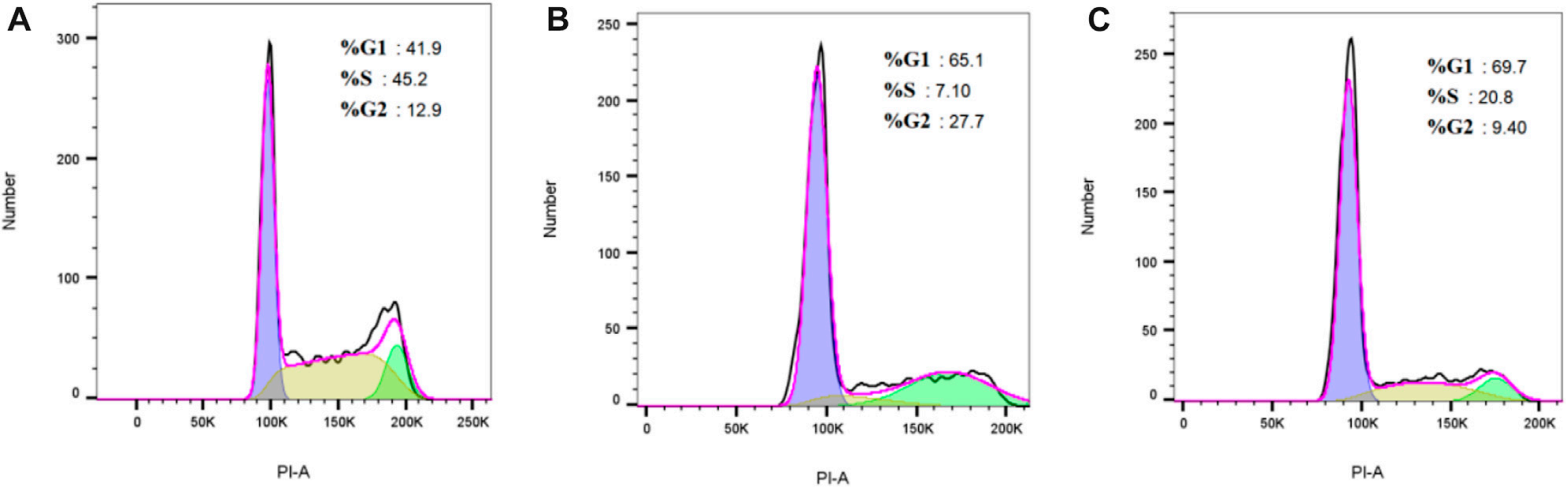
**(A–C)** Were the cell cycle of SUP-B15 cells under different administration concentrations of Imatinib, Dasatinib, and flumatinib **(A)** Negative control group; **(B)** Flumatinib 3.12 μmol/L; **(C)** Flumatinib 1.56 μmol/L.

Due to the inhibitory effect of flumatinib on cell proliferation, the effect of flumatinib on cell cycle was detected using cell loss apparatus. The results revealed the increased proportion of G1 phase cells in SUP-B15 cells after flumatinib was treated with SUP-B15 cells, as the most obvious in 3.12 μmol/L group, suggesting the SUP-B15 block by flumatinib in G1 phase (Figure 11).

## 4 Discussion

### 4.1 Clinical efficacy and adverse reactions of flumatinib

A total of 29 patients with Ph+ ALL were enrolled in the present study. After the therapy of flumatinib combined with VP induction, the CR rate reached 96.3%, with 3-month CR rate of 87.5%, and 6-month of 86.7%, which showed a slight superiority to A Phase II Study of Flumatinib with Chemotherapy for Newly Diagnosed Ph/BCR-ABL1-Positive Acute Lymphoblastic Leukemia in Adults (Liu et al., 2022). The remission rate was comparable to previous studies on Imatinib and Dasatinib (Daver et al., 2015; Ravandi et al., 2016; Foà and Chiaretti, 2022), suggesting a good effect of flumatinib on the remission rate in Ph^+^ ALL patients, comparable to second-generation TKI. However, the long-term survival rate of flumatinib in Ph^+^ ALL patients requires further observation by follow-up.

Over the years, monitoring MRD has been adopted as a key element in acute leukemia management to more precisely define the depth of patient response to treatment, including Ph+ ALL (Nishiwaki et al., 2016). The MRD conversion time of patients in the present study showed the negative conversion rate of MRD in 1, 2, and 3 months after treatment of 82.6%, 91.3%, and 95.6%, respectively. These results suggested a good effect of flumatinib on promoting the negative conversion of MRD.

In terms of molecular genetics, Ravandi scholars (Ravandi et al., 2010) suggested the remised Ph+ ALL promoted by TKI. In the present study, the MMR rates reached 73.9%, 87.5%, and 93.3% in January, March, and June after using flumatinib combined with chemotherapy, and 52.2%, 62.5%, and 73.3% for CMR rates, respectively. It is suggested a promotive role in quantitative reduction of the BCR-ABL fusion gene by flumatinib even after flumatinib combined with chemotherapy, accompanied by the increased the MMR rate between groups with the extension of treatment time, which confirms the continuous effect of flumatinib on the molecular remission effect in Ph+ ALL patients.

The remission rates of patients receiving flumatinib at first diagnosis and those changed from other TKI to flumatinib were analyzed, which revealed no statistical difference. It suggested an identify efficacy by flumatinib even on those change from other TKI due to adverse drug reactions or ABL kinase mutation. However, considering the confounding factors in the comparison of the two groups of patients, such as different combination chemotherapy, different mutant spectra, the presence of extramedullary disease or the receipt of alloHSCTs, this univariate analysis still has some limitations.

The efficacy and prognosis suggested age as a significantly factor to impact the efficacy of molecular remission of the disease. In terms of prognosis, age ≥60 years old, presence of IKZF1, failure to achieve induced remission or MMR after 3 months of treatment, and the failure to undergo hematopoietic stem cell transplantation in high-risk patients will result in adverse effects on prognosis. During treatment, it mainly referred to myelosuppression, liver insufficiency and infection, and the observed reactions such as cardiotoxicity and renal impairment were generally tolerable.

This study refers to a retrospective analysis depending on a single center with a relatively limited number of cases, and various confounding factors inevitably affect the results. In the later stage, it is necessary to expand the sample size and conduct a multi-center randomized controlled trial to further evaluate the difference in efficacy of imatinib, dasatinib and flumatinib in patients with Ph+ ALL.

### 4.2 Detection of blood concentration of flumatinib

The pharmacokinetic (PK) of flumatinib indicated that flumatinib could serve as a safe and well tolerantly therapy, with the maximum blood concentration (Cmax) and AUC0-t linearly correlated to the dose range of 200–1,000 mg (Luo et al., 2010). Studies (Jiang et al., 2023) have indicated a rapid effect by flumatinib after oral administration, with a median serum peak time (Tmax) of 2 h in CML patients and 400 mg or 600 mg of Flumatinib mesylate tablets, with the maximum concentration (Cmax) of 36.67 ± 12.95 ng/mL and 63.58 ± 51.60 ng/mL, respectively, and the half-life (t1/2 value) of 16.0–16.9 h. In phase 1 (Aishen, 2010) and unpublished phase 2 studies (TKI for CML-CP patients), plasma concentrations reached a stable state in 6–8 days after continuous administration of flumatinib, with the molecular response positively correlated with plasma concentrations of flumatinib valley.

In the present study, the TKI concentrations in peripheral blood, bone marrow, and cerebrospinal fluid were collected from 11 patients receiving flumatinib, 3 patients receiving dasatinib, and 2 patients receiving imatinib and analyzed, revealing the highest by flumatinib in Ph+ ALL bone marrow, ordered as bone marrow > serum > cerebrospinal fluid. In contrast, the concentration of dasatinib and imatinib in blood was ordered as serum > bone marrow > cerebrospinal fluid. The wide distribution of flumatinib in tissues suggests a more considerable scavenging effect on intramedullary and extramedullary infiltrated leukemia cells. Meanwhile, that flumatinib could be detected in the cerebrospinal fluid of Ph+ ALL patients suggests a high probability to cross the blood-brain barrier, which requires study with the extended data considering the relatively limited number of patients in this study. The concentration of dasatinib in the cerebrospinal fluid of the patients was lower than the lowest limit of detection, which means a requirement of further study to investigate the correlation between dasatinib and the treatment of central leukemia.

### 4.3 Pharmacodynamic experiment of SUP-B15

Pharmacodynamic analysis of Ph+ ALL cell line SUP-B15 revealed the differentiation elicited by flumatinib, as well as the promoted apoptosis of SUP-B15 cells, inhibited proliferation of Ph+ ALL cells, and the block of cells in the G1 phase. Moreover, the IC50 value reached the lowest by flumatinib in the CCK-8 compared with imatinib and dasatinib, suggesting a better effect of flumatinib.

In summary, flumatinib could achieve a better remission effect on Ph+ ALL, with comparable efficacy to second-generation TKI, and resulting in tolerable adverse reactions. Cytological experiments can also support the above conclusions. In terms of drug concentration monitoring, flumatinib was revealed to penetrate the blood-brain barrier, which is worth promoting in clinic.

## Data Availability

The raw data supporting the conclusion of this article will be made available by the authors, without undue reservation.
